# A novel fluoride tray-assisted workflow for artificial intelligence-driven automated maxillary gingival segmentation on cone beam CT

**DOI:** 10.1093/dmfr/twag023

**Published:** 2026-05-14

**Authors:** Dhanaporn Papasratorn, Rellyca Sola Gracea, Rocharles Cavalcante Fontenele, Reinhilde Jacobs

**Affiliations:** OMFS-IMPATH Research Group, Department of Imaging and Pathology, Faculty of Medicine, KU Leuven, Leuven 3000, Belgium; Department of Oral and Maxillofacial Surgery, University Hospital Leuven, Leuven 3000, Belgium; Department of Oral and Maxillofacial Radiology, Faculty of Dentistry, Mahidol University, Bangkok 10400, Thailand; OMFS-IMPATH Research Group, Department of Imaging and Pathology, Faculty of Medicine, KU Leuven, Leuven 3000, Belgium; Department of Oral and Maxillofacial Surgery, University Hospital Leuven, Leuven 3000, Belgium; Department of Dentomaxillofacial Radiology, Faculty of Dentistry, Universitas Gadjah Mada, Yogyakarta 55281, Indonesia; OMFS-IMPATH Research Group, Department of Imaging and Pathology, Faculty of Medicine, KU Leuven, Leuven 3000, Belgium; Department of Oral and Maxillofacial Surgery, University Hospital Leuven, Leuven 3000, Belgium; Department of Stomatology, Public Oral Health and Forensic Dentistry, Division of Oral Radiology, School of Dentistry of Ribeirão Preto, University of São Paulo, Ribeirão Preto 14040-904, Brazil; OMFS-IMPATH Research Group, Department of Imaging and Pathology, Faculty of Medicine, KU Leuven, Leuven 3000, Belgium; Department of Oral and Maxillofacial Surgery, University Hospital Leuven, Leuven 3000, Belgium; Department of Dental Medicine, Karolinska Institute, Stockholm 141 04, Huddinge, Sweden

**Keywords:** gingiva, segmentation, cone beam CT, artificial intelligence, deep learning

## Abstract

**Objectives:**

To clinically validate an artificial intelligence (AI)-based tool for automated maxillary gingival segmentation of marginal and supracrestal gingiva on cone beam CT (CBCT) scans using a novel soft tissue separation technique with a fluoride tray.

**Methods:**

A validation set of 35 CBCT scans, covering maxilla, acquired with fluoride trays was processed using a cloud-based AI platform (Relu, Leuven, Belgium) for gingival segmentation. The resulting models were refined by an expert using 3-dimensional (3D) mesh-processing software and compared with the original AI outputs to assess accuracy. Additionally, 6 CBCT scans were manually segmented, using intraoral scans as reference, and compared with the AI model. Surface-based and voxel-wise analyses, color-coded maps, consistency, and time-efficiency were evaluated. Wilcoxon signed-rank test was used to assess time differences among methods.

**Results:**

Artificial intelligence vs expert refinement showed strong agreement with high overlap (medianDSC ≥ 96%) and minor surface deviations (medianMSD∼0.00 mm). Minor differences were found between anterior and posterior regions (medianΔDSC = 1%, ΔMSD∼0.00 mm). Artificial intelligence vs manual segmentations showed median dice similarity coefficient (DSC) of 82% and small median MSD of 0.15 mm. Labial/buccal area showed the highest surface deviations from color-coded maps. Bland-Altman plots showed low intra- and inter-operator consistency in time, while AI showed excellent consistency. Artificial intelligence demonstrated significantly faster segmentation, achieving 4x faster with expert refinement and 20x faster with manual approach.

**Conclusion:**

The use of fluoride tray facilitates separation of oral soft tissues on CBCT scans, enabling accurate and time-efficient AI-driven segmentation of maxillary marginal and supracrestal gingiva. This supports integration into digital workflows and more efficient treatment planning.

**Advances in knowledge:**

The use of a fluoride tray for soft tissue separation during CBCT scans facilitates gingival visualization while maintaining patient comfort. The resulting 3D gingival models from AI-based segmentation can be integrated with automatically segmented dentomaxillofacial structures, enhancing clinical visualization of oral soft and hard tissues and facilitating diagnosis and treatment planning, including periodontal evaluation, implant planning, prosthodontics, and orthodontics.

## Introduction

The gingiva plays a crucial role in achieving successful dental treatment outcomes and optimal aesthetics, especially in the region visible during speech and smiling. Accurate assessment of the gingiva has become a crucial step in achieving long-lasting restorations, implant success, and favorable outcomes in orthodontic procedures.^1^^,^^2^ Gingival evaluation typically relies on clinical examination, such as assessing the gingival biotype and thickness. However, these methods can be invasive, involving transgingival probing, and subjective.^3–5^

Cone beam CT (CBCT) has become a valuable tool for assessing gingival thickness through cross-sectional images.^6^^,^^7^ To visualize gingival contours on CBCT scans, soft tissue separation techniques, such as cotton rolls or lip retractors, have been employed to distinguish the gingiva from adjacent mucosa.^8^^,^^9^ Additionally, the registration of intraoral scans (IOS) into the digital workflow further supports gingival assessment, as previous studies have demonstrated strong agreement between IOS-based and clinical measurements of gingival thickness.^10–12^

**Figure 1:**
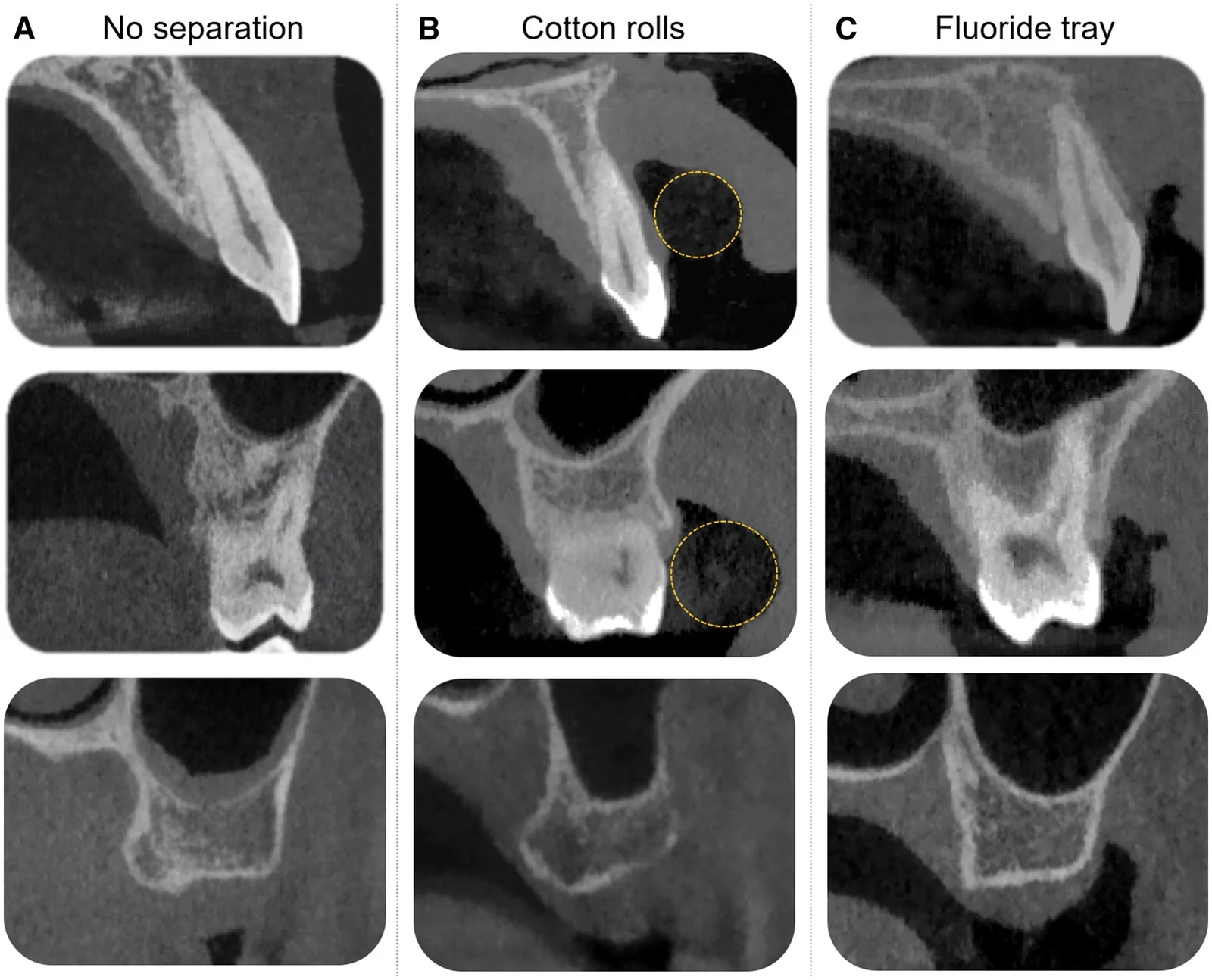
CBCT scans of the maxillary anterior region, posterior regions, and tuberosity obtained without soft tissue separation (A), with cotton rolls (B), and with a disposable fluoride tray (C). Yellow circles indicate the relative radiodensity of the cotton rolls.

Despite these advantages, the cotton roll separation technique may cause discomfort for patients and raise hygiene concerns for saliva contamination in CBCT settings. In addition, the relative radiodensity of cotton rolls may interfere with image interpretation, as illustrated in Figure 1B. Most commercially available cotton rolls are not composed of pure organic cotton; instead, they consist of compressed cotton blended with polyester or other synthetic fibres to enhance strength and absorbency. Placement of multiple rolls on both the vestibular and lingual aspects may displace the tongue toward the palate, thereby obscuring the palatal gingival contour and compromising soft-tissue delineation, in addition to increasing the likelihood of patient movement during CBCT scanning. Comparable limitations are observed with lip retractors, which may offer inadequate separation in the molar region and are often associated with increased patient discomfort or pain. Furthermore, registration between IOS and CBCT images can be challenging and prone to error, particularly in cases with extensive edentulous areas or pronounced metallic artifacts.^13^^,^^14^

The use of a disposable fluoride tray offers an appealing alternative to cotton rolls, as it is easy to position, generally comfortable for patients, and commonly available in most dental clinics. When viewed on cross-sectional CBCT images, the fluoride tray represents minimal to undetectable radiodensity (Figure 1C), reducing visual interference at the gingival margin. The separation boundary included the marginal and supracrestal gingiva, which are crucial regions for gingival assessment. This separation enables more accurate delineation of the gingival contour for segmentation, especially in the maxillary anterior region where aesthetic demands are high. Additionally, the use of fluoride tray allows isolation of the soft tissue at the maxillary tuberosity, where placement of cotton rolls or a lip retractor is difficult or impossible. This allows visualization of potential donor sites where both soft and hard tissues may be harvested for grafting procedures (Figure 1C, third row). When combined with artificial intelligence (AI) methods, particularly deep learning and convolutional neural networks (CNNs), which enable rapid and accurate image segmentation,^15–21^ this fluoride tray-assisted workflow shows strong potential to support AI-driven automated 3-dimensional (3D) gingival segmentation.

Therefore, this study aimed to validate an AI-based tool for automated maxillary gingival segmentation of the marginal and supracrestal gingiva on CBCT scans acquired using a novel oral soft tissue separation technique with a fluoride tray. The hypothesis was that the AI model would achieve accuracy comparable to expert performance, with superior consistency and time efficiency compared to the manual approach.

## Methods

This study was conducted in accordance with the World Medical Association Declaration of Helsinki principles for medical research ethics. The Local Institutional Ethics Board granted ethical approval (reference number: S69363). Scans were obtained from patients referred to the Dentomaxillofacial Imaging Centre for justified clinical indications, independent of the present study. Informed consent was obtained from all patients and from the guardians of adolescent patients prior to insertion of the fluoride tray, and all patient data were fully anonymized.

### Dataset

A total of 35 CBCT scans were retrieved from the hospital database for AI validation. The patients were 18 females and 17 males with an average age of 36 ± 24 years. In addition, 6 IOS acquired with the Trios 3 IOS (3Shape, Copenhagen, Denmark) were available and retrieved. The CBCT scans were obtained from 2 devices: NewTom VGi Evo (Cefla, Imola, Italy) and 3D Accuitomo 170 (J Morita, Kyoto, Japan). The scanning parameters for the NewTom VGi Evo were 110 kilovoltage peak (kVp), 3-20 milliampere (mA), with fields of view (FOVs) of 8 × 8, 10 × 10, 12 × 8, 16 × 16, and 24 × 19 cm, and voxel sizes of 0.125-0.300 mm. For the 3D Accuitomo 170, the parameters were 90 kVp, 5 mA, with FOVs of 8 × 8 and 10 × 10 cm, and voxel sizes of 0.160-0.250 mm.

**Figure 2:**
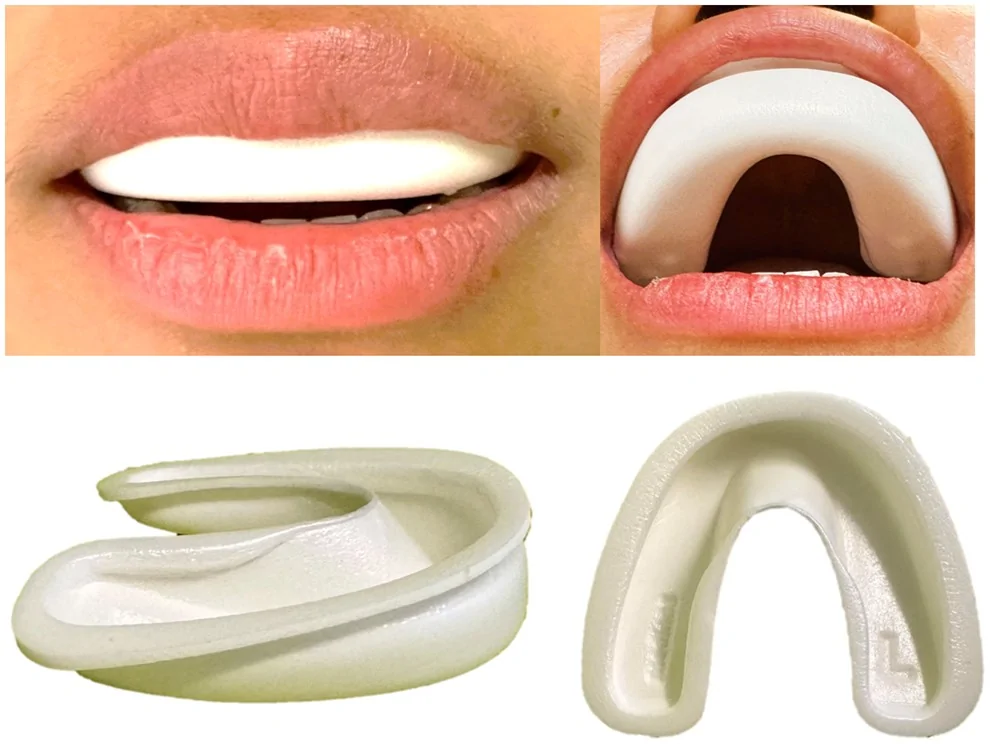
Fluoride tray with the intraoral view placed in the maxillary arch to aid soft tissue separation during CBCT scans.

This study used disposable fluoride trays on the maxillary arch solely for research purposes. The use of a fluoride tray did not cause any additional scatter or increase the risk of patient motion; therefore, there was no additional radiation dose exposure. All examinations were performed in accordance with the standard radiation protection protocols of the Dentomaxillofacial Imaging Centre to ensure dose minimization. Tray sizes (medium or large) were selected according to the dimensions of each patient’s maxillary arch (Yellow Point, Polo MB, Oisterwijk BV, The Netherlands). The medium size was employed for early adolescents with mixed dentition, while the large size was used for late adolescents and adults. The fluoride trays are made of foam with a soft texture, allowing easy insertion into the maxillary arch to separate the lip from the buccal gingiva and the tongue from the anterior hard palate. Images of the fluoride tray are shown in Figure 2.

Inclusion criteria consisted of CBCT scans that covered the entire maxillary arch with a fluoride tray in place during image acquisition. Exclusion criteria included scans with motion blur or pronounced beam-hardening artifacts that obscured the gingival contours.

### AI-based gingival segmentation

Automated gingival segmentation was performed using Relu Creator (Relu, Leuven, Belgium), a cloud-based AI-assisted platform for dental and maxillofacial imaging. The underlying CNN for gingival segmentation was based on a 3D U-Net architecture optimized for dentomaxillofacial CBCT segmentation and implemented on this cloud platform in a beta version, which is not yet commercially available. The gingival segmentation model has been clinically validated in a prior study.^22^

**Figure 3:**
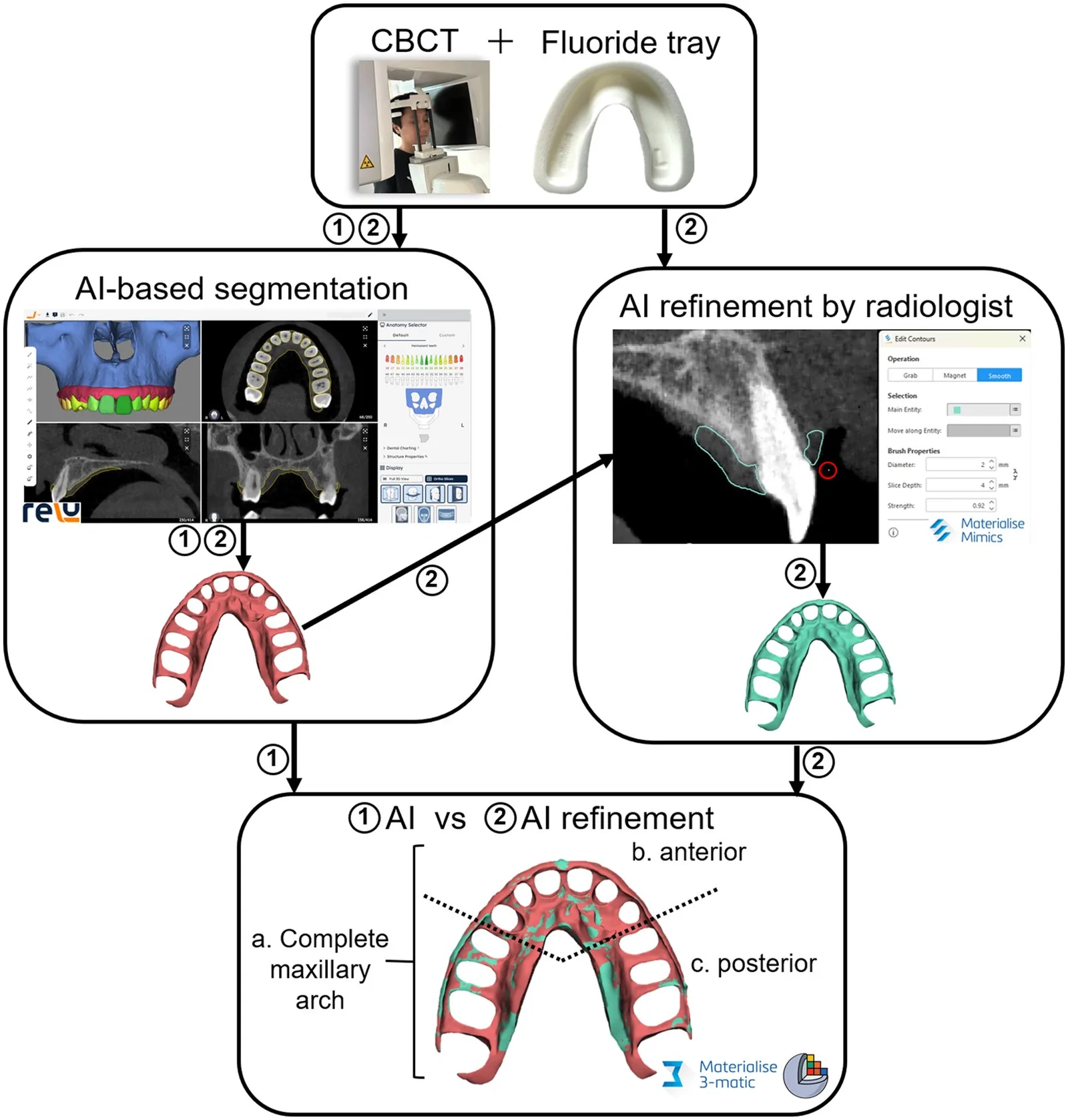
Workflow for AI-based and refined AI gingival segmentation approaches. Abbreviations: AI = artificial intelligence; CBCT = cone beam CT.

The workflow for each patient consisted of the following steps: selecting the Virtual Patient AI module, uploading the CBCT scan in Digital Imaging and Communications in Medicine (DICOM) format, and recording the time taken. Time was tracked throughout the entire process, from starting the upload to finishing, including opening patient files, selecting the maxillary gingiva, and exporting the results. The AI-based workflow panel is shown in Figure 3.

### Refinement of an AI-based gingival segmentation

Maxillary gingival segmentation refinement was performed by an oral and maxillofacial radiologist (D.P.) using Mimics software (version 25.0, Materialise, Leuven, Belgium) and independently reviewed by a second radiologist (R.S.G.). Any discrepancies were discussed and resolved by consensus, with adjustments made directly in the software. Figure 3 illustrates the refined AI workflow. The AI-segmented gingiva was exported in the stereolithography (STL) format and imported into Mimics, along with the corresponding CBCT scan. The Edit Contours 3D tool was primarily used to adjust the contour of the maxillary gingiva on multiplanar views of the CBCT scans. Refinements were made by delineating the gingival contours, which were visible on CBCT scans. The time it took to complete the refinement process was recorded from start to finish, ending with the final export of the refined gingiva STL file.

### Manual gingival segmentation

Manual segmentation was performed independently by 2 oral and maxillofacial radiologists (D.P. and R.S.G.) on 6 CBCT scans using IOS data as a reference for more precise manual gingival delineation. This was performed using Mimics and 3-matic software (version 17.0, Materialise, Leuven, Belgium), following a protocol established in a previous validation study.^22^ The CBCT and IOS data were uploaded to the ReLU Creator, where the IOS were automatically aligned with the CBCT scan, as validated by Elgarba et al.^23^ Previously validated segmented maxillary teeth, skull, and registered IOS were exported in STL format for further processing.^15^^,^^16^^,^^24^^,^^25^

**Figure 4:**
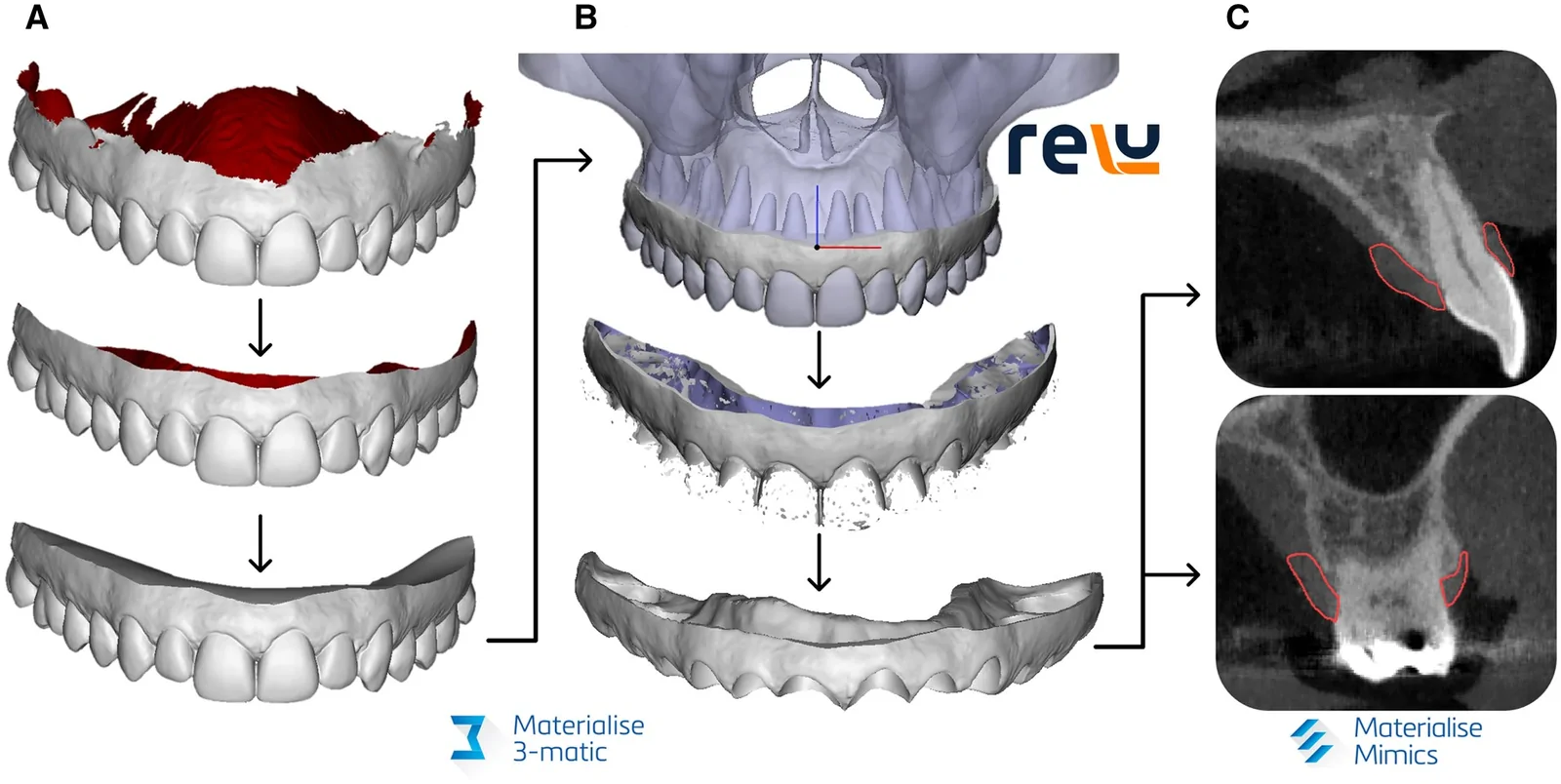
Workflow of manual segmentation: (A) creation of a solid model, (B) subtraction of skull and teeth, and removal of excess areas, then (C) delineation of the gingival contour to match the outline visible on the CBCT scan with fluoride tray separation.

In the 3-matic software, the outer borders of the registered maxillary IOS were trimmed and filled with a freeform-shaped surface to create closed solid models (Figure 4A). The segmented skull and teeth were then imported, and the “Subtract tool” was used to remove the hard tissue areas of these structures. The remaining excess areas were manually trimmed and smoothed into a gingiva shape (Figure 4B). The resulting gingiva and its corresponding CBCT scan were then imported into the Mimics software program (Figure 4C). Gingival contours were delineated to match the visible outline on CBCT images acquired with the fluoride tray separation, using the “Edit Contours 3D tool.” This procedure was performed similarly to the refinement workflow of the AI-based gingival segmentation. Figure 4 illustrates the overall workflow of the manual approach.

The 2 operators independently performed the segmentation process, and the main operator (D.P.) repeated the task within a 2-week interval. The agreement between the resulting segmentation models of the complete maxillary gingiva was then evaluated to assess intra-operator consistency across repeated segmentations and inter-operator consistency between the 2 operators. The time duration started when the CBCT and IOS data were imported into the segmentation software and ended upon exporting the manually segmented gingiva.

### Evaluation of AI-based gingival segmentation

For the quantitative evaluation, the point-based surface distance between the AI-based gingival segmentation and the refined-AI segmentation was assessed using the Part Comparison tool in 3-matic software. The evaluation included 3 metrics: median surface distance (MSD), root mean square distance (RMSD), and Hausdorff distance (HD).^26^ The voxel-based evaluation between the AI-based and refined-AI gingival segmentation was performed using the Segment Comparison module, an installed extension in SlicerRT in the 3D Slicer image computing platform (version 5.9.0, slicer.org, Slicer Community).^27^ The dice similarity coefficient (DSC) and Jaccard Index (JAC) were calculated to assess the overlap between the 2 segmented models.^28^ These metrics were calculated for the complete maxillary arch, as well as for its anterior and posterior regions. The maxillary arch was divided into anterior (canine to canine) and posterior (premolars and molars) regions using a reference plane positioned at the distal surfaces of the canines, as shown in Figure 3. This analysis focused on both quantifying discrepancies and measuring overlap between the AI segmentation and the radiologist’s refinement. The segmentation times were also compared between the segmentation approaches.

Qualitative analysis involved evaluating color-coded deviation maps obtained from the Part Comparison tools. These maps illustrated areas of surface differences and were assessed by an oral and maxillofacial radiologist (D.P.) on a standard monitor screen (Dell U2415b UltraSharp 24-in Widescreen LCD, Dell, Round Rock, TX, United States). Using the segmentation analysis tools in 3-matic software, the gingival surfaces were assigned colors based on the deviation range from −1.00 mm under-segmentation (blue color) to +1.00 mm over-segmentation (red color). The surface region (labial/buccal, proximal, or palatal) exhibiting the largest deviation area, or the greatest number of red or blue-colored regions, was identified, and its frequency was recorded across the complete maxillary arch as well as in the anterior and posterior segments.

### AI-based vs manual gingival segmentation

Six maxillary gingival models generated by the AI-based approach were compared with manual segmentations to assess accuracy using the same metrics as for the aforementioned analysis: MSD, RMSD, HD, DSC, and JAC. For each case, the manual reference was jointly selected by both radiologists. Additionally, the 2 segmentation approaches were compared in terms of the time required to complete the segmentation and the consistency of segmentation times across repeated segmentations.

### Statistical analysis

Statistical analyses were conducted using RStudio version 2025.05.1 + 513 (Posit, Boston, United States). The selected accuracy metrics (MSD, RMSD, HD, DSC, JAC) were first evaluated for normality using the Shapiro-Wilk test. Since the results indicated a non-normal distribution (*P *< .05), descriptive statistics were summarized using medians, upper and lower quartiles, and minimum and maximum values. Non-parametric tests were applied for inferential statistics.

The non-parametric, 2-sided Wilcoxon signed-rank test was employed to analyze the differences in average time among all segmentation methods. A *P*-value of less than .05 was deemed statistically significant. Additionally, inter- and intra-operator consistency for the time required to perform manual segmentation was evaluated using Bland-Altman plots.

## Results

### AI-based vs refinement of an AI-based gingival segmentation

Descriptive statistics relative to the selected metrics considering the complete maxillary gingiva are presented in Table 1. For complete maxillary gingiva, the median values of MSD, RMSD, and HD were 0.00 mm (interquartile range [IQR]: 0.00-0.01 mm), 0.20 mm (IQR: 0.14-0.37 mm), and 2.31 mm (IQR: 1.42-3.65 mm), respectively. The DSC was ≥ 96% and JAC ≥ 93%.

**Table 1:** Comparison of evaluated metrics between AI-based and refinement of AI-based segmentation of the complete maxillary gingiva.

	Maxillary arch (AI vs refined-AI)				
Metric	Min	Q1	Median	Q3	Max
MSD [mm]	0	0	0	0.01	0.06
RMSD [mm]	0.07	0.14	0.2	0.37	0.72
HD [mm]	0.85	1.42	2.31	3.65	6.54
DSC (%)	84	95	96	97	99
JAC (%)	73	90	93	95	98

Abbreviations: DSC = dice similarity coefficient; HD = Hausdorff distance; JAC = Jaccard Index; MSD = median surface distance; Q1 = lower quartile; Q3 = upper quartile; RMSD = root mean square distance.

The results for the anterior and posterior regions of the maxillary gingiva are presented in Table 2. Overall, segmentation performance was comparable across regions, with only minor differences.

**Table 2:** Comparison of evaluated metrics between AI-based and refinement of AI-based segmentation of the anterior and posterior maxillary gingiva.

Metric	Region	Min	Q1	Median	Q3	Max
MSD (mm)	Anterior	0.00	0.00	0.00	0.02	0.10
	Posterior	0.00	0.00	0.00	0.01	0.05
RMSD (mm)	Anterior	0.07	0.13	0.21	0.32	1.09
	Posterior	0.04	0.10	0.18	0.26	0.64
HD (mm)	Anterior	0.66	1.24	1.81	2.48	6.54
	Posterior	0.58	1.12	1.85	3.19	5.08
DSC (%)	Anterior	81	94	96	97	99
	Posterior	89	96	97	98	100
JAC (%)	Anterior	68	89	93	94	97
	Posterior	80	91	94	97	99

Abbreviations: DSC = dice similarity coefficient; HD = Hausdorff distance; JAC = Jaccard Index; MSD = median surface distance; Q1 = lower quartile; Q3 = upper quartile; RMSD = root mean square distance.

In the anterior region, the median MSD, RMSD, and HD were 0.00 mm (IQR: 0.00-0.02 mm), 0.21 mm (IQR: 0.13-0.32 mm), and 1.81 mm (IQR: 1.24-2.48 mm), respectively. The corresponding median DSC and JAC were 96% and 93%.

In the posterior region, the median MSD was 0.00 mm (IQR: 0.00-0.01 mm), RMSD was 0.18 mm (IQR: 0.10-0.26 mm), and HD was 1.85 mm (IQR: 1.12-3.19 mm). The median DSC and JAC were 97% and 94%, respectively.

**Figure 5:**
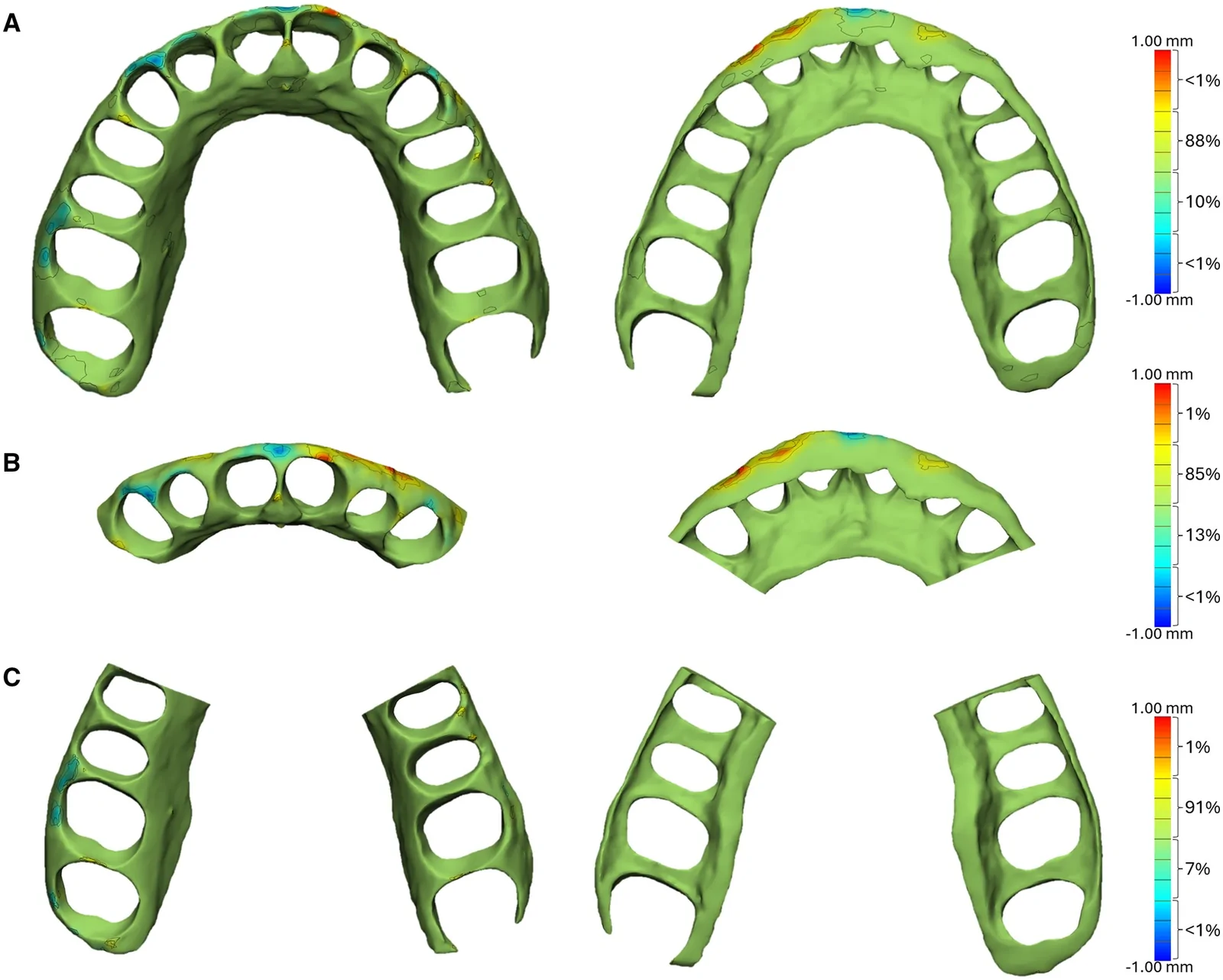
Color-coded maps of AI-based segmentation, showing surface discrepancies with radiologist refinement from −1.00 mm under-segmentation (blue color) to +1.00 mm over-segmentation (red color) for (A) complete maxillary gingiva, (B) anterior, and (C) posterior regions. The histogram analysis indicates that green areas (differences within 0.25 mm) cover ≥96% of the surface.

Figure 5 shows color-coded maps illustrating the discrepancies between the AI-based and refined-AI gingival segmentations. Considering the complete maxillary gingiva, most discrepancies occurred on the labial surfaces of the anterior teeth, followed by the proximal areas of the posterior teeth. In the anterior region, discrepancies were mainly seen on the labial surfaces of the incisors, where the AI-based segmentation tended to be thicker and extended more apically toward the roots of the maxillary teeth. In the posterior region, discrepancies were mainly observed on the buccal surfaces of the second premolars and the molar area, followed by the proximal gingiva. The number of sites exhibiting the maximum discrepancy per region and surface is summarized in Table 3.

**Table 3:** Number of sites showing the maximum discrepancy per region and tooth surface based on histogram analysis of color-coded maps.

Surface	Entire maxillary arch (*n*)	Anterior region (*n*)	Posterior region (*n*)
Labial/buccal	20	23	17
Proximal	12	6	15
Palatal	3	6	3

### AI-based vs manual gingival segmentation

**Figure 6:**
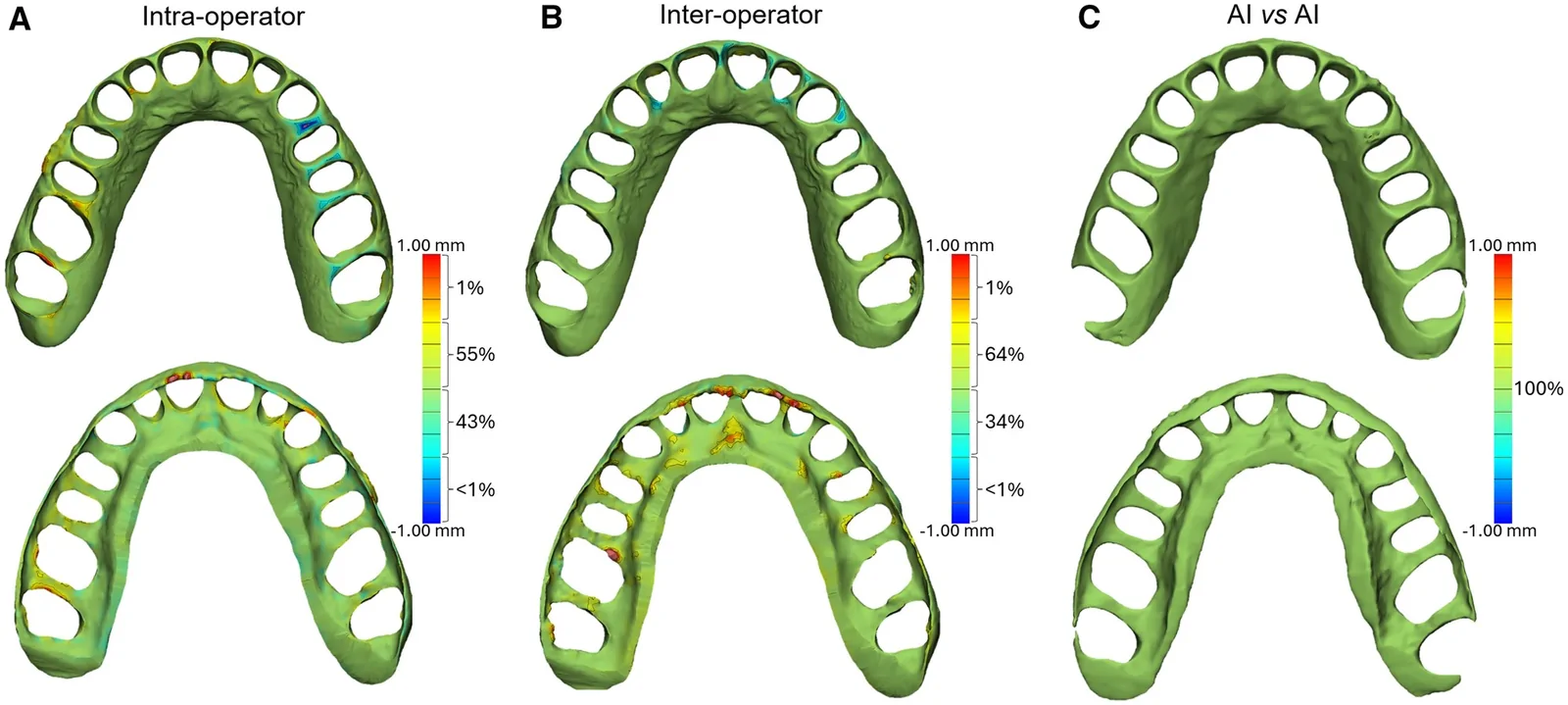
Color-coded maps showing surface discrepancies (−1.00 to +1.00 mm) between (A) intra-operator, (B) inter-operator, and (C) AI *vs* AI segmentations. AI segmentation showed incomplete segmentation in the buccal region of the second molars. Histogram analysis indicates that green areas (differences within 0.25 mm) cover ≥93% of the surface, except for AI, which demonstrated perfect consistency.

Table 4 presents the descriptive statistics of AI vs manual segmentation of the complete maxillary arch. The median values of MSD, RMSD, and HD were 0.15 mm (IQR: 0.14-0.16 mm), 0.46 mm (IQR: 0.38-0.57 mm), and 3.10 mm (IQR: 2.18-3.88 mm), respectively. The median DSC was ≥ 82% and JAC ≥ 70%. For intra-operator comparison, the median values of MSD, RMSD, and HD were 0.02 mm (IQR: 0.01-0.03 mm), 0.16 mm (IQR: 0.13-0.28 mm), and 1.51 mm (IQR: 1.47-2.62 mm), respectively. The median DSC was ≥ 94%, and JAC was ≥ 89%. Inter-operator comparison also yielded comparable values, with the median values of MSD, RMSD, and HD being 0.02 mm (IQR: 0.01-0.02 mm), 0.17 mm (IQR: 0.15-0.27 mm), and 2.14 mm (IQR: 1.71-2.57 mm), respectively. The median DSC was ≥ 95% and JAC ≥ 91%. Figure 6 displays color-coded maps comparing the segmentation consistency of intra-operator, inter-operator, and AI segmentations. Histogram analysis revealed that the largest surface deviations in manual segmentations occurred along the labial flanges, whereas AI segmentations showed no differences between repeated runs.

**Table 4:** Descriptive statistics of AI vs manual segmentations, intra-operator, and inter-operator segmentations.

	Maxillary arch					
Comparisons	Metrics	Min	Q1	Median	Q3	Max
Manual vs AI	MSD (mm)	0.13	0.14	0.15	0.16	0.18
	RMSD (mm)	0.34	0.38	0.46	0.57	0.66
	HD (mm)	1.81	2.18	3.1	3.88	3.97
	DSC (%)	76	80	82	84	86
	JAC (%)	61	66	70	73	75
Intra-operator (manual segmentation)	MSD (mm)	0.00	0.01	0.02	0.03	0.05
	RMSD (mm)	0.11	0.13	0.16	0.28	0.44
	HD (mm)	1.34	1.47	1.51	2.62	3.59
	DSC (%)	91	93	94	96	96
	JAC (%)	83	87	89	91	93
Inter-operator (manual segmentation)	MSD (mm)	0.00	0.01	0.02	0.02	0.05
	RMSD (mm)	0.13	0.15	0.17	0.27	0.37
	HD (mm)	1.34	1.71	2.14	2.57	3.85
	DSC (%)	91	94	95	96	96
	JAC (%)	83	88	91	92	92

Abbreviations: DSC = dice similarity coefficient; HD = Hausdorff distance; JAC = Jaccard Index; MSD = median surface distance; Q1 = lower quartile; Q3 = upper quartile; RMSD = root mean square distance.

The Wilcoxon signed-rank test showed a statistically significant difference between the total AI-based time and the radiologist’s refinement time (*P *< .01), as well as between the AI-based time and the manual segmentation (*P *< .01). The average ± standard deviation of total time for AI-based gingival segmentation, including CBCT scan uploading time, was 287 ± 127 s. Excluding upload time, the segmentation time from selecting the patient’s case to downloading the segmented gingiva was 26 ± 9 s. The average time for the radiologist refinement was 1183 ± 420 s and for manual segmentation was 5660 ± 213 s. On average, AI-based gingival segmentation was 4 times faster than radiologist refinement and approximately 20 times faster than the manual approach, demonstrating substantial time efficiency.

**Figure 7:**
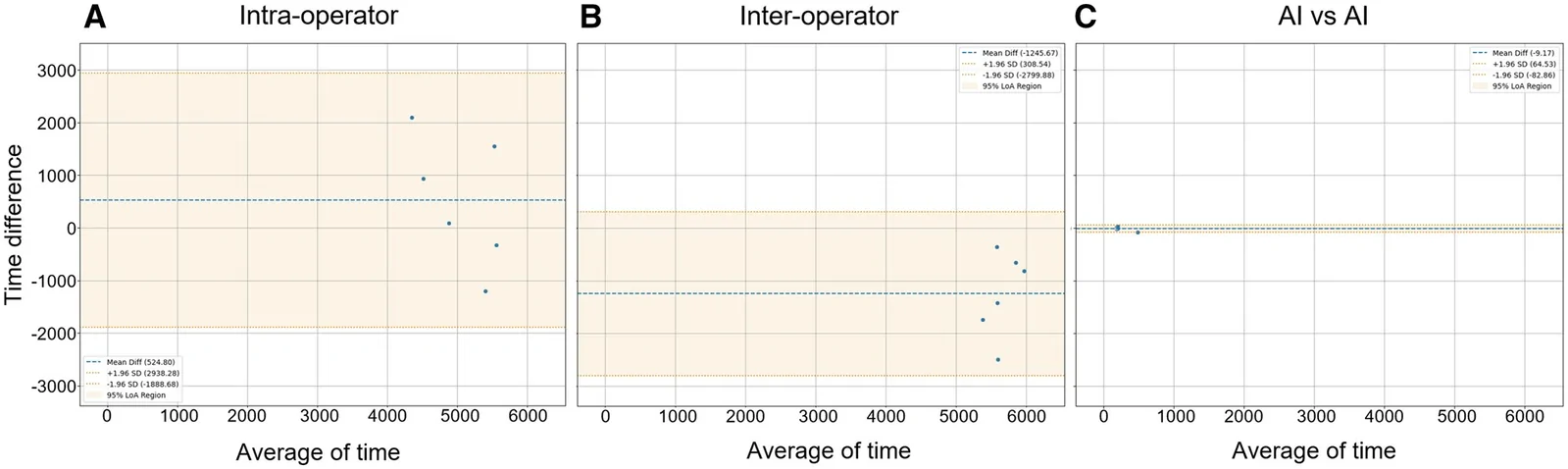
Bland-Altman plots of (A) intra-operator, (B) inter-operator, and (C) AI *vs* AI segmentation time comparisons, presented on the same axis scale for direct comparison. Each point represents 1 of the 6 selected CBCT scans for manual segmentation. The dashed blue horizontal lines indicate the mean difference (bias), while the dashed orange lines indicate the 95% limits of agreement (±1.96 SD). AI showed both excellent time efficiency and consistency, with tight clustering of all points and short average segmentation times. Abbreviation: AI = artificial intelligence.

To assess the time consistency of segmentation, Figure 7 presents the Bland-Altman plots for intra-operator, inter-operator, and AI segmentations. For the intra-operator analysis (D.P.1 vs D.P.2), all differences between the 2 manual segmentation times were within the limits of agreement (LoA; −1889 to 2938 s). However, the LoA was wide, and several data points were far from the mean difference line, indicating low consistency for the same operator. The inter-operator analysis (average D.P. vs R.G.) also showed low consistency, but with a narrower LoA (−2800 to 309 s). In contrast, the AI segmentation demonstrated excellent agreement, with a narrow LoA from −83 to 65 s, indicating highly time-consistent performance.

## Discussion

Given the increasing capabilities of AI in medical imaging, this study presents a novel approach that uses a disposable fluoride tray to separate soft tissue during CBCT scanning, enabling automated maxillary gingival segmentation directly from CBCT scans. This separation technique yielded highly accurate segmentation of the marginal and supracrestal gingiva, with only minor discrepancies compared to the refined models performed by the oral and maxillofacial radiologists. These findings highlight the AI model’s robustness and suggest high generalizability for AI-based gingival segmentation that was separate by different technique.

The fluoride tray provides better hygiene, patient comfort, and time-savings compared to cotton rolls or lip retractors. It also inherently separates the tongue from the anterior hard palate, which cannot easily be achieved with inverted lip retractors, as these require patients to push their tongues toward the floor of their mouths.^29^ Fluoride trays create more natural conditions than cotton rolls and lip retractors for visualizing the gingiva as a whole. A minimal degree of separation, together with a thin interposed air layer, is sufficient to allow gingival segmentation. This method enables adequate separation of the marginal and supracrestal gingiva, which are critical regions for the assessment of clinical parameters such as gingival biotype and thickness, and facilitates measurement of the distance from the gingival margin to the labial/buccal alveolar crest and the cemento-enamel junction.^30^^,^^31^ These assessments are particularly valuable for accurately assessing gingival morphology, aesthetic treatment planning, and smile design.^32–37^

The 3D gingival models obtained in the present study demonstrate a strong potential to enhance the assessment of gingiva morphology and contour. When combined with the platform’s validated tooth and bone segmentation, the resulting anatomical reconstruction shows a promising ability to represent a 3D periodontal model, supporting more accurate diagnosis and treatment planning.^15^^,^^16^^,^^23^^,^^24^^,^^38^^,^^39^ However, this has not yet been achieved, as the model cannot integrate the limited information that CBCT cannot provide, such as gingival color or inflammation. Compared with conventional CBCT-IOS superimposition methods, the AI-derived gingival models allowed visualization of both gingival contour and thickness, rather than representing only the gingival outline captured by the IOS. This can enhance evaluation of the gingival biotype, which is critical for a wide range of dental procedures, including implant planning, smile design, and orthodontic treatment.^40^

The AI-based segmentation yielded strong performance for the complete maxillary gingiva as well as for its anterior and posterior regions. To assess the surface differences between AI-based and refined 3D gingival models, this study utilized 3 metrics: MSD, RMSD, and HD. Median surface distance reflects the typical discrepancy, RMSD captures the impact of outliers and more pronounced discrepancies, and HD identifies the maximum observed deviation between surfaces. Additionally, both DSC and JAC were used to assess segmentation overlap. Although these are mathematically related, DSC is more sensitive to overlapping regions, whereas JAC provides a stricter evaluation, penalizing small mismatches more heavily. Together, they are considered the 2 most common measures of region overlap.^28^^,^^41^

The current study indicated that the median and maximum MSD values between AI and expert refinement (*n* = 35) remained under 0.10 mm across all regions, slightly outperforming the results reported for maxillary structures, which ranged from 0.25 to 0.49 mm.^17^^,^^42^ The MSD values are also comparable to those from a previous study using cotton rolls for soft tissue separation (maxillary gingiva 0.07 mm).^22^ These variations fall within the typical CBCT voxel size (0.125-0.40 mm), suggesting that they are not clinically meaningful.^20^^,^^43^^,^^44^ The differences in MSD between the anterior and posterior regions were minimal. Both regions had median MSD values below 0.01 mm, confirming the low clinical impact of the observed differences. Root mean square distance values were also within acceptable limits, with median values below 0.21 mm. Median DSC and JAC were both higher than 93%, indicating high segmentation performance and similarity to the 3D gingival model generated after refinements.

Higher maximum RMSD and HD values between AI and expert refinement (*n* = 35) indicated some localized areas of more variability. These were mainly found at the apical edges of the labial gingiva in the anterior region and extended beyond the fluoride tray’s coverage area. It should be emphasized that the fluoride tray was designed to enhance soft tissue separation at the marginal and supracrestal gingiva. Therefore, the increased deviations observed at the apical level fall outside the intended separation boundary and require future validation to determine whether they are due to the AI system detecting gingival structures based on subtle radiodensities that are not easily visible to the human eye.^45–47^

Color-coded deviation maps revealed that over-segmentation predominantly occurred on the labial surfaces of anterior teeth, especially near the incisors. In these areas, the AI-segmented gingiva mostly extended beyond the separation boundary of a fluoride tray on CBCT scan, corresponding to the regions with the highest RMSD and HD values. In the posterior region, discrepancies were most pronounced on the buccal surfaces. These findings are consistent with a previous study indicating that AI models face challenges in detecting soft tissue boundaries.^18^^,^^48^ Similar limitations have been reported in tooth and periodontal ligament segmentation studies, where regions with intricate anatomical structures presented reduced accuracy.^49^ However, the median discrepancies across all evaluated metrics were generally minimal and remained clinically insignificant.

AI-based gingival segmentation demonstrated performance comparable to expert annotations (*n* = 6), with a high voxel-wise overlap (median DSC = 82%) and a small median surface deviation (median MSD = 0.15 mm), indicating that these differences are below the typical CBCT voxel size and are not clinically significant.^20^^,^^43^^,^^44^ Intra- and inter-operator consistency were high, with median DSC above 94% and median MSD of 0.02 mm, confirming the reliability of the AI refinement for validation purposes. When AI is compared with expert refinement (*n* = 35), the median DSC increased to 96%, and the median MSD reached 0.00 mm, demonstrating that minimal expert intervention can achieve near-perfect segmentation.

The AI model demonstrated a remarkable time efficiency, completing segmentation approximately 4 times faster than expert refinement and 20 times faster than the manual approach. This substantial reduction in processing time highlights the potential of AI tools to enhance clinical workflows and reduce clinician workload.^17^^,^^20^^,^^21^^,^^24^^,^^25^^,^^28^ Furthermore, AI demonstrated excellent time consistency, unlike human performance, which showed low consistency as observed in Bland-Altman plots.

Several limitations should be considered in this study. First, the sample size was limited. However, it is comparable to an independent test dataset used to validate the performance of a pre-trained AI model to evaluate generalizability.^28^^,^^48^^,^^37^ Second, although disposable fluoride trays are available in multiple sizes, the largest size may still be insufficient to fully cover the maxillary second molars or tuberosity in some adults, potentially limiting segmentation accuracy in that region. Another consideration is the limitation of radiologist refinement, which depends on visual interpretation of the regions with clearly distinguishable gingival boundaries. In contrast, the AI model can detect subtle grayscale variations and may segment gingival margins beyond the separation boundary of the fluoride tray, which radiologists cannot always confirm in some cases.^45–47^ In addition, the absence of soft tissue color information on CBCT remains a limitation for comprehensive periodontal evaluation, particularly for distinguishing inflammation or subtle gingival changes.^32^^,^^33^^,^^50^ Simultaneous placement of fluoride trays in both arches reduces patient comfort and may compromise stability during CBCT scanning, consequently increasing the likelihood of motion artifacts occurring. Therefore, future investigations are required to optimize the tray design, or alternatively, to conduct dedicated validation focusing specifically on the mandibular gingiva.

Future validation of this automated gingival segmentation approach should include applications to the mandibular arch and patients with various clinical conditions. It would also be valuable to assess its performance in cases with pronounced artifacts from high-density materials, as these areas remain challenging for clinicians to evaluate accurately. Moreover, studies exploring longitudinal changes in gingival contours using automated segmentation may offer insights into treatment outcomes or disease progression. Despite these considerations, the current findings demonstrate that automated segmentation of the maxillary gingiva is highly effective with minimal, clinically insignificant error. This highlights its strong potential for generalization and clinical utility.

## Conclusion

The use of a fluoride tray represents a novel approach to facilitating soft tissue visualization on CBCT scans. Combined with AI-driven segmentation, this method offers highly accurate, consistent, and time-efficient delineation of maxillary marginal and supracrestal gingiva, demonstrating strong potential for integration into digital clinical workflows. This approach showed promising potential to develop into a 3D periodontal model to facilitate diagnosis and treatment planning through visualization of hard and soft tissues.
